# Research of Fog Seal Performance with Sand Materials for Airport Asphalt Pavements

**DOI:** 10.3390/ma18174050

**Published:** 2025-08-29

**Authors:** Hui Zhang, Zhe Hu, Yongsheng Guan, Dongliang Hu

**Affiliations:** 1 Jiangsu Sinoroad Engineering Research Institute Co., Ltd., Nanjing 211805, China; zh@sinoroad.com (H.Z.); gys@sinoroad.com (Y.G.); 2 School of Highway, Chang’an University, Xi’an 710064, China; hu_chd@126.com

**Keywords:** airport asphalt pavement, sand-containing fog seal, spraying amount, surface properties, durability

## Abstract

Asphalt pavements are widely used in airports due to their excellent skid resistance, vibration damping, and ease of construction. However, traditional fog seal materials often suffer from insufficient adhesion between fine sand and the emulsified asphalt binder, resulting in limited durability of the maintenance effect. This study aims to optimize the design of traditional fog seal materials and systematically evaluate their surface and durability performance. Firstly, a composite modified emulsified asphalt was prepared as the sand suspension slurry for the sand-containing fog seal. Through the dry wheel abrasion test, the optimal fine aggregates content was determined for four different spraying amounts (0.8, 0.9, 1.0, and 1.1 kg/m^2^). When the proportion of fine aggregates increases, the spraying amount needs to be increased accordingly to ensure the wrapping effect. Subsequently, pavement performance evaluation was conducted based on several indicators, including surface curing time, British Pendulum Number (BPN) friction coefficient, permeability coefficient, and mass loss rate. The results showed that the designed sand-containing fog seal significantly reduced surface curing time and exhibited superior skid resistance and permeability property compared to styrene-butadiene rubber (SBR)-modified emulsified asphalt. After freeze–thaw cycles, the maximum decrease in friction coefficient was 10.2%, and the mass loss rate after abrasion was approximately 67%, which were lower than those of SBR-modified emulsified asphalt (22.2% and 81%, respectively). Finally, considering the comprehensive performance comparison and evaluation, the optimal mix proportion was determined as 1.0 kg/m^2^ spraying amount with 30% fine aggregates content. The findings of this study provide practical support for improving the durability and service life of airport asphalt pavements.

## 1. Introduction

As an integral part of modern comprehensive transportation systems, civil aviation plays an irreplaceable strategic role in promoting regional economic coordination and facilitating international exchange and cooperation [1,2]. Airport pavements, as critical infrastructure within the civil aviation operational system, directly support aircraft taxiing, take-off, and landing, and thus form the core foundation for ensuring the safety and efficiency of air transport [3,4]. Asphalt concrete pavements are widely adopted in airports worldwide due to their excellent surface smoothness, ease of construction, and convenient maintenance [5]. However, under the combined effects of long-term repeated loading and environmental factors, asphalt pavements may be susceptible to various distresses such as surface abrasion, rutting, and cracking, which may ultimately threaten the stable and safe operation of aircrafts [6,7]. With the global increase in newly built airports, the demand for maintenance of asphalt pavements in airports is rising rapidly, and pavement maintenance has become a key area of development [8]. Therefore, it is imperative to adopt scientifically effective maintenance strategies for asphalt pavements to extend service life, reduce life-cycle maintenance costs, and enhance operational safety [9].

Proactive and preventive maintenance have been proven to be an effective approach to delay the pavement performance deterioration and reduce the occurrence of distresses [10]. Proactive maintenance can promptly address issues that arise during the use of airport pavements, ensuring the normal operation of runways [11]. For example, runway cleaning can prevent foreign objects from interfering with aircraft takeoff and landing, repairs can be carried out on damaged areas, and friction testing can ensure runway skid resistance, thereby enhancing flight safety. Moreover, commonly used preventive maintenance techniques for asphalt pavements include micro-surfacing, crushed stone seal, fog seal, and various ultra-thin overlays [12,13]. Compared with highway asphalt pavements, airport asphalt pavements are subjected to greater operational loads and more frequent repeated impact loads [14,15]. In addition, depending on geographic location and elevation, they may also be exposed to extreme climatic factors, such as high and low temperatures, rainfall, snowfall, and freeze–thaw cycles [16]. Due to the special functional requirements of runways, even minor structural or surface distresses can pose direct safety risks to aircraft operations, making timely preventive maintenance essential [17]. Fog seal is a widely applied preventive maintenance technique in China’s highway and airport asphalt pavements, valued for its simple construction, quick reopening to traffic, and long-term effectiveness [18,19]. It can effectively fill micro-cracks and surface voids, mitigate asphalt aging, and prevent aggregates raveling [20]. However, the traditional emulsified asphalt commonly used as the raw materials in fog seal often suffers from performance deficiency, including low mechanical strength, insufficient adhesion, limited waterproof capability, and inadequate durability [21]. In addition, fog seal applications may present limitations, such as a reduction in pavement friction and insufficient abrasion resistance after application [22]. For example, Islam et al. [23] tested fog seal materials in field applications and found that the materials could fully cure within 2.5 to 3.5 h, reducing hydraulic conductivity by an average of 38.5%, but it also caused a 20–40% decrease in the friction coefficient. The consistency of the material can also affect its penetration depth into the pavement, thereby impacting crack repair effectiveness and other functions. Moreover, Li et al. [24] found that for the emulsified asphalt as matrix additive fog sealer, its evaporation residue content was high, the adhesion performance was poor, and the stripping rate exceeded 75% under low-temperature conditions.

To enhance the adhesion of fog seal and improve pavement skid resistance, researchers have proposed sand-containing fog seal technology that incorporates fine sand, fine aggregates, and various additives into the slurry [25]. Applied as a thin layer using specialized spraying equipment, this technique not only extends pavement service life, but also improves the performance of the road surface [26]. Additionally, scholars have developed various functionalized sand-containing fog seal materials, such as composite modified emulsified asphalt, bio-based fog seal, and silicone resin fog seal, and have improved durability by optimizing mix proportions, aggregates gradation, and adding thickeners [27,28]. For example, Cai et al. [21] prepared a novel composite fog seal material from emulsified asphalt and waterborne epoxy system, and found that the spraying amount of 0.6–0.7 kg/m^2^ with the mixture consisting of 100% modified emulsion, 25–40% sand, and 8% cement in weight ratio, the material achieved the high adhesion strength and very low permeability, while having minimal impact on the friction coefficient. Zhang et al. [29] investigated the permeability and skid resistance of fog seal, showing that pore structures were more easily filled as the spraying amount increased, significantly reducing the permeability. Moreover, the reduction in surface texture depth also led to lower skid resistance, and a spraying amount of 0.2–0.4 kg/m^2^ was recommended to balance the skid resistance and waterproof performance. Ma et al. [30] optimized fog seal material proportions and studied the effects of water, mineral fillers, and sand content on curing time, viscosity, adhesion, and abrasion resistance by using the response surface methodology, and suggested that fine aggregates content could be kept below 25% to improve abrasion resistance and bonding strength. Jiang et al. [31] conducted experiments on the relationship between the ratio of sand to emulsified asphalt, coating rate, and abrasion resistance in fog seal layers, and the results showed that adding the approximately 1:1 ratio resulted in a 27.4% decrease in the friction coefficient after 100,000 abrasion cycles and demonstrated good quality loss control. Factors such as spraying amount, sand content, and fine aggregates proportion play critical roles in improving the durability of sand-containing fog seal [32]. However, the existing research mainly focuses on optimizing single performance aspects and lacks systematic exploration of multi-performance and engineering applicability in airport asphalt pavements. Therefore, it is necessary to comprehensively balance sand content, fine aggregates dosage, and preparation process to develop a high-performance sand-containing fog seal with enhanced skid resistance, abrasion resistance, and impermeability, providing the technical reference for extending airport asphalt pavement service life and improving maintenance effectiveness.

The main objective of this paper is to optimize the design and comprehensively evaluate the performance of sand-containing fog seal materials for asphalt pavements used in airports. Firstly, a composite modified emulsified asphalt was prepared as the sand suspension slurry to produce the sand-containing fog seal materials. Through the dry wheel abrasion test, the optimal fine aggregates content under four different spray rates was systematically analyzed. Additionally, a two-way analysis of variance (ANOVA) was employed to explore the effects of spraying amount and fine aggregates content on abrasion resistance. Subsequently, the prepared sand-containing fog seal materials were comprehensively evaluated by five aspects, including surface curing performance, skid resistance, abrasion resistance, impermeability, and freeze–thaw durability. Finally, the optimal ratio of sand-contained fog seal with superior performance in key indicators including surface curing time, British Pendulum Number (BPN), pavement permeability coefficient (*C_w_*), and mass loss rate was selected, providing technical reference for improving pavement performance and extending the service life of airport asphalt pavements.

## 2. Materials and Methods

### 2.1. Raw Materials

The emulsified asphalt was prepared using 90# matrix asphalt and the slow-breaking, fast-setting, fatty amide-based polyamine cationic emulsifier (SL-MK538) produced by Shanghai Songli New Materials Technology Co., Ltd., Shanghai, China, with an emulsifier content of 1.6%. In this study, an emulsified asphalt with 60% solid content was prepared. The specific preparation steps are as follows (taking 1000 g of emulsified asphalt as an example): Firstly, 600 g of the required matrix asphalt, 16 g of emulsifier, and 384 g of distilled water were weighed, the distilled water was added to a container containing the emulsifier, heated to 60 °C, and stirred evenly, adjusting the soap solution pH to 2–3. Then, the matrix asphalt was heated to 160 °C and added to a colloid mill together with the prepared soap solution for high-speed shear emulsification. Finally, the emulsified material was collected, transferred, and mixed according to the set procedure to ensure uniform and stable emulsified asphalt. The emulsified asphalt was tested based on the MH/T 5011-2019 [33], and the specific test results are shown in Table 1.

The waterborne epoxy resin (WER) system consisted of WER emulsion (bisphenol-A type epoxy emulsion, 0.25 mol/100 g epoxy equivalent) and curing agent (aliphatic amine), with a dosage ratio of 1:1.3 between WER emulsion and the curing agent, and 15% of the WER system was added to the emulsified asphalt for modification, following the findings of Yang et al. [34]. Additionally, 5% styrene-butadiene rubber (SBR, 25% styrene content) was incorporated to enhance the low-temperature toughness of the emulsified asphalt. Garnet sand (140 μm average particle size and 6.6 m^2^/g specific surface area) and sodium bentonite (12 μm average particle size and 77.2 m^2^/g specific surface area) were selected as fine aggregates and thickener, respectively. Other additives used included a defoamer (polyether-modified polydimethylsiloxane organosilicon, AFE-1410), a surfactant (alkylphenol ethoxylate, TX-10), and a dispersant (sodium polyacrylate). Finally, to promote the curing of the SBR/WER-modified emulsified asphalt, Portland cement (P.O 42.5) was also added.

### 2.2. Samples Preparation

**Figure 1:**
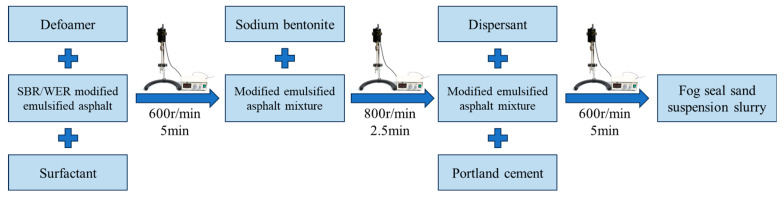
The preparation of fog seal sand suspension slurry materials.

The durable seal fog with sand material was prepared using SBR/WER composite modified emulsified asphalt as the binder, combined with the addition of surfactant, fine aggregates, thickener, defoamer, dispersant, and cement. All additive dosages are expressed as mass ratios relative to the composite modified emulsified asphalt as shown in Table 2. The specific preparation process of the sand suspension slurry can be illustrated in Figure 1. The stirring temperature was room temperature (25 °C), and all additives were stirred for the same duration of 2.5 min [35]. Firstly, 1% surfactant and 0.8% defoamer were sequentially added to the SBR/WER emulsified asphalt and mixed using a digital display stirrer manufactured by Shanghai Lichen Instrument Technology Co., Ltd. (Shanghai, China) at a speed of 600 r/min for each 2.5 min. This step ensures that the surfactant and defoamer are fully dispersed in the slurry while eliminating any potential bubbles and foam. Then, 15% pre-weighed sodium bentonite was slowly added in the slurry, followed by stirring at 800 r/min for 2.5 min. When adding the thickener, the stirrer should be turned off to prevent the thickener from sticking to the inner wall of the container and to ensure its better solubility, thus achieving a uniform distribution in the slurry. Finally, 2% dispersant and 3% cement were sequentially added and mixed at 600 r/min for each 2.5 min. The dispersant can improve the dispersion of liquid modifiers and delay the agglomeration of liquid materials.

The sand-containing fog seal was sprayed onto compacted asphalt concrete rutting plates (300 mm × 300 mm × 50 mm). After the rutting plates were dried and cooled to room temperature, spraying was carried out. The spraying equipment was fitted with a fan-shaped nozzle, with a spraying pressure of 0.30 MPa, a nozzle-to-sample surface distance of 20 cm, and a moving speed of 0.1 m/s to ensure uniform coverage. The environmental conditions during spraying were 25 ± 2 °C and 60 ± 5% relative humidity. After spraying, the specimens were cured under the same environmental conditions. In this study, four different spraying amounts were considered, 0.8 kg/m^2^, 0.9 kg/m^2^, 1.0 kg/m^2^, and 1.1 kg/m^2^, along with five fine aggregates contents of 15%, 20%, 25%, 30%, and 35%; the selected ranges of spraying amounts and fine aggregate contents were based on the following references [32,36,37]. A total of 20 types of seal fog with sand material samples were prepared, with their abbreviations shown in Table 3. For example, the sample with the spraying amount of 0.8 kg/m^2^ and 15% fine aggregates content is denoted as S1F1, while the sample with the spraying amount of 1.1 kg/m^2^ and 35% fine aggregates content is denoted as S4F5.

### 2.3. Experiments and Evaluation Indicators

#### 2.3.1. Dry Wheel Abrasion Test

The abrasion resistance was evaluated by studying the differences in abrasion values of sand-containing fog seal materials under varying fine aggregates contents and spraying amounts through the dry wheel abrasion test conducted by the SYD-0752 abrasion tester, following the method in the JT/T 1330-2020 [38]. Initially, a silicone mold with a central circular hole of 280 mm diameter was prepared. An asphaltic felt disc was placed on the test bench, and the silicone mold was carefully positioned on top of it. Then, 300 g of the sand suspension slurry was weighed and mixed with different proportions of fine aggregates using a mixer. The blended materials were poured into the silicone mold and leveled with a scraper. After molding, the samples were placed in a 60 °C oven and dried to a constant weight. Once fully cured, the samples were removed from the oven, cooled, and the combined mass of the asphaltic felt and samples were recorded as (*M*_1_). Finally, the tray containing the samples was placed steadily on the abrasion tester platform. After starting the device and waiting for 5 min, the device was closed, and the samples and asphaltic felt disc were removed and placed back into the 60 °C oven for drying. After cooling, the total mass of the asphaltic felt and samples was measured again and recorded as (*M*_2_). The abrasion value of the sand-containing fog seal materials was calculated using Equation (1).

(1) $$WTAT=\frac{M_{1}-M_{2}}{A}$$

where *WTAT* is the dry wheel abrasion value of the sand-containing fog seal materials (kg/m^2^); *M*_1_ is the total mass of the oil felt and samples before abrasion (g); *M*_2_ is the total mass of the oil felt and specimen after abrasion (g); and *A* is the abrasion area of the rubber tube of the abrasion head (*A* = 0.0279 m^2^).

#### 2.3.2. Surface Dryness Test

Based on the optimal combination of fine aggregates content determined from the dry wheel abrasion test under four different spraying amounts, the surface dryness test was conducted using rutting plates with reference to the JT/T 1330-2020 [38]. The SBR/WER emulsion sand-containing fog seal materials were applied onto rutting plates specimens, with conventional SBR-modified emulsified asphalt used as the control group (spraying amount of 0.9 kg/m^2^). The specimens were cured at 25 °C, and the surface condition was observed and recorded every 0.5 h. Finally, the surface curing time of the SBR/WER emulsified asphalt sand-containing fog seal materials at each optimal combination was determined. In this test, a brown paper towel was gently pressed on the specimen surface every 30 min to check its dryness. The test was considered complete when no water stains appeared on the paper towel.

#### 2.3.3. Skid Resistance Test

In airport pavement evaluation, the skid resistance of asphalt pavements is typically characterized by texture depth and friction coefficient. In this study, asphalt concrete rutting plates (300 mm × 300 mm × 50 mm) were used, and the skid resistance test was conducted by the BM-III pendulum tester with reference to the JTG 3450-2019 [39], and the evaluation index was the BPN. Firstly, the initial BPN of the rutting plates without any surface treatment was recorded. Then, four kinds of fog seal materials with optimal mix ratios were applied, along with a conventional emulsified asphalt fog seal as a control group, ensuring the same spraying amounts. The changes in skid resistance before and after applying the sand-containing fog seal were then analyzed.

#### 2.3.4. Permeability Test

According to the requirements for the permeability coefficient specified in the MH/T 5010-2017 [40], the permeability test was carried out by the pavement water seepage meter. The SBR/WER emulsified asphalt sand-containing fog seal with different fine aggregates contents were prepared using the optimal mix ratios and uniformly applied onto rutting plate specimens. After curing, the following steps were performed: firstly, a plastic ring was positioned at the center of the rutting plate. Then, a metal ring was placed over the plastic ring for sealing, and the seepage meter was positioned so that it fitted snugly into the metal ring. Next, the valve was closed, the measuring cylinder was filled with water, and then the valve was opened so that water began to flow downward gradually. Finally, the water level in the measuring cylinder was recorded every 10 min, and the test was stopped after 1 h. The specific seepage coefficient was then calculated using Equation (2).

(2) $$C_{w}=\frac{V_{2}-V_{1}}{T_{2}-T_{1}}$$

where *C_w_* is the pavement permeability coefficient (mL/min); *V_1_* and *V_2_* are the values of initial and final values of water level, respectively (mL); and *t_1_* and *t_2_* are the corresponding time, respectively (min).

#### 2.3.5. Accelerated Wear Test

The abrasion resistance of the SBR/WER emulsion sand-containing fog seal under the optimal design mix was evaluated by the mass loss rate (*F_a_*) of the rutting plates through the Three-Wheel Accelerated Wear Tester. The specific procedure of the accelerated wear test can be seen as follows: Firstly, preparing the rutting plates specimens and recording their initial mass as m_b_. Then, applying four optimal combinations of sand-containing fog seal onto the specimens and measuring the mass after coating, recorded as m_a_. Afterwards, placing the specimens into the accelerated wear tester, and ensuring that they are securely fixed to prevent uneven abrasion during the test, and recording the specimen mass before abrasion as m_1_. Finally, starting the tester to carry out abrasion on the sand-containing fog seal specimens. After reaching the set number of abrasion cycles, the mass of the specimens after abrasion was recorded as m_2_. The value of F_a_ can be calculated according to Equation (3).

(3) $$F_{a}=\frac{m_{1}-m_{2}}{m_{a}-m_{b}}\times \frac{A_{s}}{A_{c}}$$

where *F_a_* is the mass loss rate (%); *m_1_* and *m_2_* are the mass of the specimens before and after abrasion (g), respectively; *m_b_* and *m_a_* are the mass of the specimens before and after applying the sand-containing fog seal (g), respectively; and *A_s_* and *A_c_* are the spreading and actual abrasion area of the sand-containing fog seal coating on the specimens (cm^2^), respectively.

#### 2.3.6. Freeze–Thaw Cycle Test

The freeze–thaw cycle test refers to the JTG E20-2011 [41]. Each test group was conducted in three replicates to ensure the reliability. The freeze–thaw resistance of the sand-containing fog seal coating at the optimal mix design was evaluated by comparing the permeability coefficient and BPN before and after the freeze–thaw cycle. The specific procedure included: Firstly, curing specimens of different sand-containing fog seal at a constant temperature of 25 °C for 24 h. Then, placing them in a −18 °C low-temperature chamber for 8 h of freezing. Next, removing the specimens and immersing them in a water tank at room temperature for 4 h of thawing, completing one freeze–thaw cycle. This cycle was repeated until a total of 10 freeze–thaw cycles were finished. Finally, the specimens were air-dried at room temperature for 12 h, and then the BPN and permeability coefficient were measured. These results were compared to the values obtained before the freeze–thaw cycles to assess the durability of the sand-containing fog seal.

## 3. Results and Discussion

### 3.1. Determination of Optimum Spraying Amount and Fine Aggregates Content

**Figure 2:**
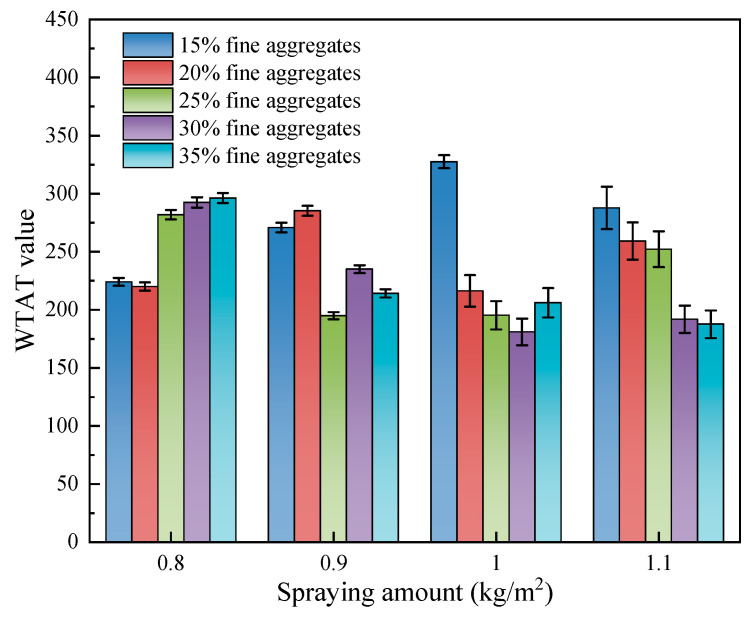
*WTAT* value of different sand fog seal materials.

The dry wheel wear test results of sand-containing fog seal materials with different spray rates and fine aggregate contents are shown in Figure 2. According to the wear resistance test standard specified in JT/T 1330-2020 [38], the *WTAT* must not exceed 350 kg/m^2^. As shown in Figure 2, under all combinations of spraying amounts and fine aggregates content, the *WTAT* values of the sand-containing fog seal can meet the specification requirements. The highest *WTAT* value 327.58 g/m^2^ was the combination of S3F1. With different combinations of fine aggregates content and spraying amount, it was found that as the spraying amount increased, the optimal fine aggregates content also increased. This indicates that a higher amount of SBR/WER emulsified asphalt slurry is needed to fully coat the fine aggregates and ensure the abrasion resistance. Specifically, the optimal fine aggregates contents corresponding to spraying amounts of 0.8 kg/m^2^, 0.9 kg/m^2^, 1.0 kg/m^2^, and 1.1 kg/m^2^ were determined to be 20%, 25%, 30%, and 35%, respectively. Among these, the combination with 1.0 kg/m^2^ spraying amount and 30% fine aggregates content achieved the lowest *WTAT*, demonstrating the best abrasion resistance.

When the spraying amount is 0.8 kg/m^2^, the *WTAT* value increases with higher fine aggregates content, indicating that within the range of 15–35% fine aggregates content, increasing fine aggregates content leads to a decrease in abrasion resistance, showing a negative correlation between abrasion resistance and fine aggregates content. Therefore, it is not recommended to use a low spraying amount when applying the sand-containing fog seal in engineering applications. The main reason is that the SBR/WER-modified emulsified asphalt cannot fully coat the excessive amounts of fine aggregates, making it difficult to improve the material’s strength. As the fine aggregates content increases from 15% to 35%, it is observed that when the spraying amount is 1.0 kg/m^2^, the *WTAT* value shows a trend of first decreasing and then increasing, reaching its minimum value when the fine aggregates content is 30%. Increasing the fine aggregates content by another 5% at this level does not improve the abrasion resistance. However, when the spraying amount is 1.1 kg/m^2^, the WTAT value gradually decreases, indicating that increasing the fine aggregates content can improve the abrasion resistance.

In addition, at fine aggregates contents of 30% and 35%, the spraying amount and *WTAT* value also show a positive correlation. This is opposite to the trend observed at lower fine aggregates contents. This can be ascribed to the reality that the higher spraying amount requires more fine aggregates to be dispersed. Therefore, when the spraying amount is higher, the *WTAT* value of the sand-containing fog seal is lower and abrasion resistance is better within the range of 15–30% fine aggregates content.

To determine the optimal fine aggregates content and spraying amount for the prepared sand-containing fog seal, a two-way ANOVA was conducted to analyze the effects and interaction of these two key factors. The main effects test results obtained from IBM SPSS Statistics 29.0.1 are shown in Table 4. In the model setup, the dependent variable was the WTAT value, while the fixed factors were fine aggregates content (five levels) and spraying amount (four levels), with an interaction term of fine aggregates content and spraying amount. The significance level for hypothesis testing was set at α = 0.05. The ANOVA output included the sum of squares, degrees of freedom, mean square, F-value, and significance level (*p*-value). A factor or interaction was considered statistically significant for the WTAT value when *p* < 0.05. The F-value was used to measure the strength of the factor’s effect on the response variable; the larger the F-value, the more significant the effect of the factor.

The results show that at a confidence interval of 0.95, the significance *p*-value for fine aggregates content (0.032) is less than 0.05, indicating that fine aggregates content has a significant effect on the *WTAT* value. In contrast, the significance *p*-value for spraying amount (0.351) is greater than 0.05, suggesting that when considered alone, the spraying amount cannot significantly affect the *WTAT* value; the influence of fine aggregates content on *WTAT* value is greater than that of the spraying amount. Moreover, the interaction between fine aggregates content and spraying amount shows an F-value of 183.0 and a significance *p*-value of 0.024 (<0.05), which means there is a significant interaction effect. Although spraying amount alone has little effect, it can significantly influence the WTAT value when combined with different fine aggregate contents. Therefore, when designing sand-containing fog seals, the interaction between fine aggregate content and spraying amount should be considered, rather than focusing solely on the effect of a single factor. Based on the dry wheel abrasion test results and two-way ANOVA analysis, the abrasion resistance of four optimal combinations can be sorted: 1.0 kg/m^2^ and 30%, 1.1 kg/m^2^ and 35%, 0.9 kg/m^2^ and 25%, and 0.8 kg/m^2^ and 20%.

### 3.2. Surface Performance Evaluation

#### 3.2.1. Surface Dryness Property

**Figure 3:**
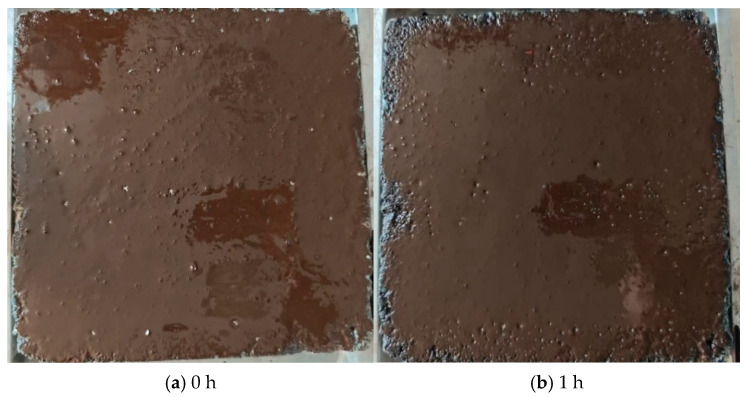

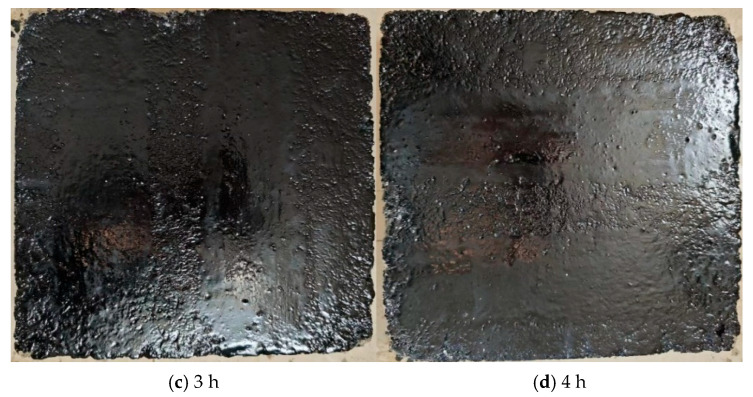
The surface dryness state of sand-containing fog seal under different curing times.

The surface curing time results for the SBR-emulsified asphalt (spraying amount of 0.9 kg/m^2^) and the four optimal SBR/WER emulsified asphalt-based sand-containing fog seal materials are shown in Table 5. Although the S1F2 exhibits the slowest surface dryness speed, it still meets the requirement of JT/T 1330-2020 that the surface curing time does not exceed 5 h [38]. Detailed observations of its curing state and surface drying process are illustrated in Figure 3.

After curing at room temperature (25 °C) for 1 h, the surface of the rutting plates still displayed uncured brown emulsified asphalt. It is in the stage of rapid water evaporation, but the SBR/WER sand-containing fog seal had not yet reached surface dryness. After 3 h curing, the materials began to fully dry on the surface. Compared to the four optimal sand-containing fog seal materials, the SBR-modified emulsified asphalt has a slightly longer surface dryness time of about 4 h. This may be due to the fact that the incorporation of waterborne epoxy resin in sand-containing fog seal can lead to chemical crosslinking. The curing process can reduce the time required for surface film formation.

Moreover, the incorporation of cement can also promote the curing of modified emulsified asphalt. In contrast, SBR-modified emulsified asphalt may mainly rely on the breaking of the emulsion to form a film without any thickener and fine aggregates, and it depends solely on evaporation of water from the surface layer. Additionally, among the four optimal combinations, the surface curing time from the fastest to slowest can be sorted: S4F5, S3F4, S2F3, and S1F2. Furthermore, the results show that the surface curing time of sand-containing fog seal decreases with increasing fine aggregates content. This may be because more fine aggregates introduce additional micro-voids in the material, which act as open pores to absorb moisture from the emulsified asphalt, thus accelerating water removal and the breaking of the emulsion, ultimately reducing the surface curing time.

#### 3.2.2. Skid Resistance

**Figure 4:**
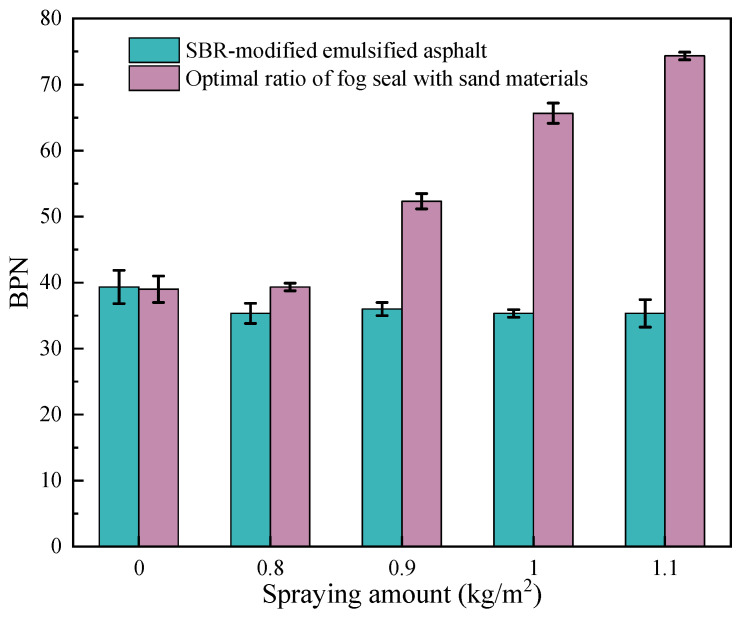
Comparison of BPN of different sand-containing fog seal materials.

The comparison of BPN results for the different fog seal materials are shown in Figure 4. Figure 4 presents that the initial BPN of the rutting plates before applying any materials was 39. When using the SBR-modified emulsified asphalt as the fog seal material, the BPN decreased slightly and then tended to stabilize as the spraying amount increased. This indicates that applying only SBR-modified emulsified asphalt did not improve the skid resistance of the original pavement surface. With further increases in the spraying amount, the skid resistance continued to decline. When the spraying amount reached 1.1 kg/m^2^, the BPN dropped to 35, representing an approximately 10% reduction compared to the BPN of the rutting plates before coating. This is because the SBR-emulsified asphalt flow penetration fills the original rough surface texture of the rutting plates.

When applying the four optimal combinations of sand-containing fog seal materials prepared in this study, the friction coefficient of the rutting plates increased to varying degrees. Notably, when applying the S1F2, the change in the BPN of the rutting plates was minimal. This may be because, under this mix ratio, most of the fine aggregates were wrapped by the waterborne epoxy sand suspension slurry and thus could not effectively contribute to skid resistance. However, when the spraying amount and fine aggregates content gradually increased, the BPN also showed a corresponding upward trend, indicating that the waterborne epoxy sand-containing fog seal can effectively enhance pavement skid resistance. This may be due to the fact that the fine aggregates in the sand-containing fog seal material increase the surface roughness and the embedding effect, and at the same time, with the increase of spraying amount, more fine aggregates can be embedded, thereby increasing the skid resistance. Among them, S4F5 can provide the best skid resistance, with the BPN increasing by 89.74% compared to the untreated rutting plates. This demonstrates that the sand-containing fog seal prepared in this study can significantly improve the original pavement’s friction coefficient compared to SBR-modified emulsified asphalt fog seal.

#### 3.2.3. Impermeability Property

According to the test results of BPN measured using the pendulum tester in Section 3.2.2, it was found that when the SBR-modified emulsified asphalt is used as the fog seal material, the skid resistance does not vary significantly regardless of the spraying amount. Moreover, an excessively high spraying amount can even reduce the abrasion resistance. Therefore, in the subsequent tests, when using the SBR-modified emulsified asphalt fog seal material as the control group, the spraying amount was fixed at 0.9 kg/m^2^, and the durability indicators under different spraying amounts of SBR-modified emulsified asphalt were no longer compared. The calculated results of the seepage coefficients for different fog seal materials are shown in Table 6. Under the application of different fog seal materials, the seepage coefficients of the rutting plates all meet the requirement of not more than 80 mL/min in the MH/T 5010-2017 [40]. When using the SBR-modified emulsified asphalt as the fog seal material, the seepage coefficient was the highest at 1.17 mL/min. In contrast, the permeability test results show that when using the sand-containing fog seal, the seepage coefficients were lower, effectively improving the impermeability property of the asphalt pavements. This is because the crosslinked three-dimensional network structure formed by the WER and curing agent can significantly reduce the permeable pathways and enhance the bonding performance of the modified emulsified asphalt [42]. Its high crosslink density enables a dense and tight sealing effect even with a relatively thin coating. Furthermore, the sand suspension slurry can penetrate into the aggregate pores of the rutting plates to supplement the open pores. Among them, the S1F2 exhibited the best impermeability.

### 3.3. Service Durability Evaluation

#### 3.3.1. Abrasion Resistance

**Figure 5:**
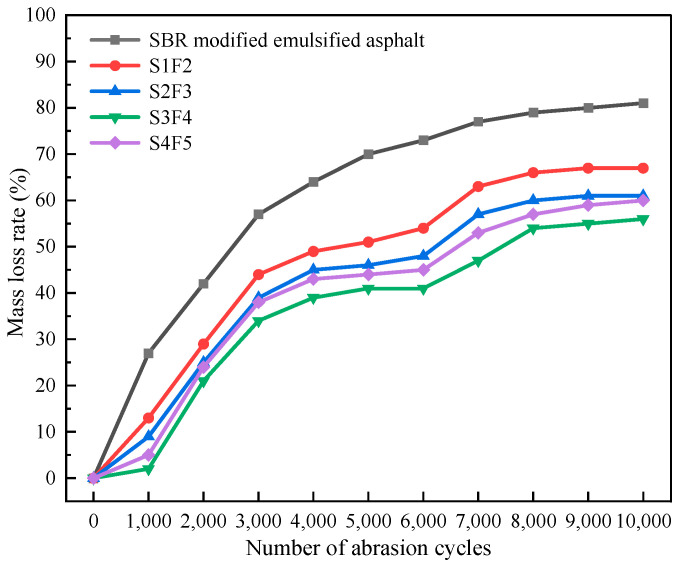
The mass loss rate curve of accelerated wear test.

The curve of mass loss rates calculated from the accelerated abrasion test for the fog seal materials is shown in Figure 5. Figure 5 shows that when using the SBR-modified emulsified asphalt as the fog seal material, the mass loss rate gradually increases with the number of abrasion cycles, reaching 70% after 5000 cycles. In contrast, for the SBR/WER emulsified asphalt sand-containing fog seal, the mass loss rates were all lower and showed three stages of stepped change, with significant inflection points observed around 3000 and 7000 cycles during the abrasion process. This change could be due to the initially faster wear rate of the surface layer with the fog seal in the early stage, leading to a rapid increase in mass loss rate. However, in the middle stage, the fine aggregates garnet sand contributed to the abrasion resistance, slowing the increase in mass loss rate. In the late stage, the sand-containing fog seals on the rutting plate surfaces were severely worn, resulting in smaller changes in the mass loss rate in the final stabilization stage. Ultimately, the mass loss rate for the SBR-emulsified asphalt fog seal material reached about 81%, while among the four kinds of sand-containing fog seal, the highest mass loss rate was 67% for the S1F2. The mass loss rate from the lowest to highest can be sorted: S3F4, S4F5, S2F3, and S1F2. When both the spraying amount and fine aggregates content were low, the bonding strength provided by the waterborne epoxy system was insufficient, the emulsified asphalt was not fully wrapped, and less fine aggregates could not form an effective skeleton structure, resulting in a relatively higher mass loss rate. In contrast, as the spraying amount and fine aggregates content increased, more SBR/WER emulsified asphalt slurry was needed to encapsulate the fine aggregates, leading to the formation of a dense and uniform skeleton structure, which could ensure better abrasion resistance of the sand-containing fog seal.

#### 3.3.2. Freeze–Thaw Resistance

**Figure 6:**
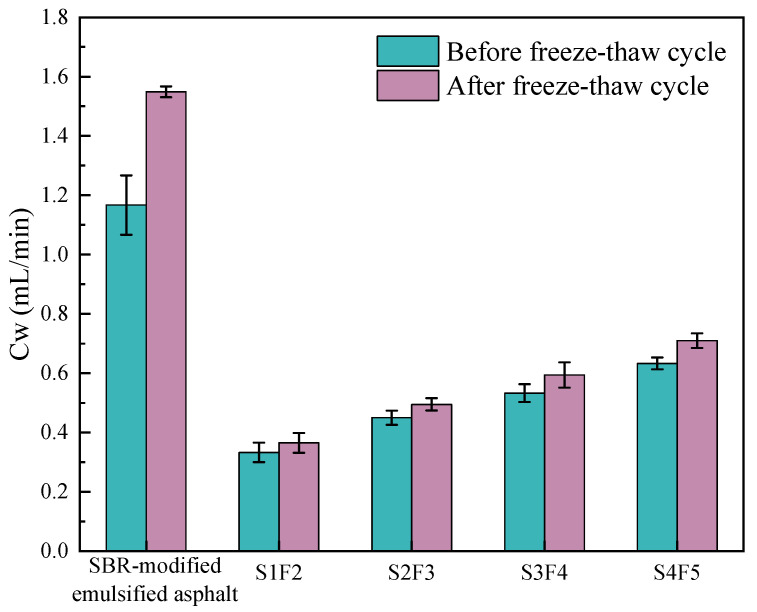
Comparison of seepage coefficient before and after freeze–thaw cycle.

The comparison of seepage coefficients before and after the freeze–thaw cycles is shown in Figure 6. Figure 6 indicates that when using the SBR-modified emulsified asphalt as the sand-containing fog seal, the seepage coefficients increased by 32.7% after the freeze–thaw cycles test. However, for the four optimal mix ratios of SBR/WER emulsified asphalt sand-containing fog seal, the increase in seepage coefficients was much smaller than that of the SBR-modified emulsified asphalt, demonstrating that the sand-containing fog seal materials have an excellent water sealing effect. A smaller seepage coefficient indicates better impermeability performance. Therefore, after the freeze–thaw cycles, the impermeability property from the strongest to weakest can be sorted: S1F2, S2F3, S3F4, and S4F5. Moreover, compared with the skid resistance test results, a lower spraying amount and fine aggregates content actually showed better impermeability properties. This suggests that when the fine aggregates content is lower, the proportion of SBR/WER emulsified asphalt slurry is higher, allowing the sand suspension slurry to penetrate the aggregates’ pores of the rutting plates and improve the overall water resistance of the fog seal. This can be explained by the fact that although the higher spraying amount and fine aggregates content can increase the surface roughness and thus enhance skid resistance, they may also lead to a higher internal void ratio. Under freeze–thaw cycles, micro-cracks and local spalling are more likely to occur, forming continuous seepage channels. Conversely, lower spraying amount and fine aggregates content can maintain the denser and uniform structure, resulting in better impermeability properties after freeze–thaw cycles.

**Figure 7:**
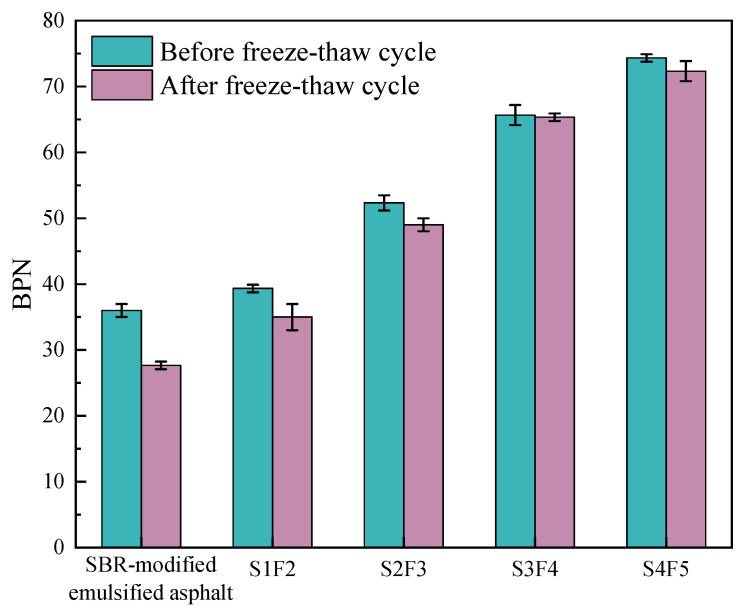
Comparison of BPN before and after freeze–thaw cycle.

In addition, the BPN results before and after the freeze–thaw cycles are shown in Figure 7. Figure 7 demonstrates that when using the SBR-modified emulsified asphalt as the fog seal material, the BPN decreased by 22.2% after the freeze–thaw cycles. In contrast, the reduction in BPN for four optimal mix ratios of sand-containing fog seal was much smaller than that of the SBR-modified emulsified asphalt. Based on the degree of BPN reduction, the freeze–thaw resistance of four optimal SBR/WER emulsified asphalt sand-containing fog seal is ranked from strongest to weakest as follows: S3F4, S4F5, S2F3, and S1F2. This is similar to the results observed in the abrasion resistance test.

### 3.4. Comprehensive Performance Comparison and Evaluation

**Figure 8:**
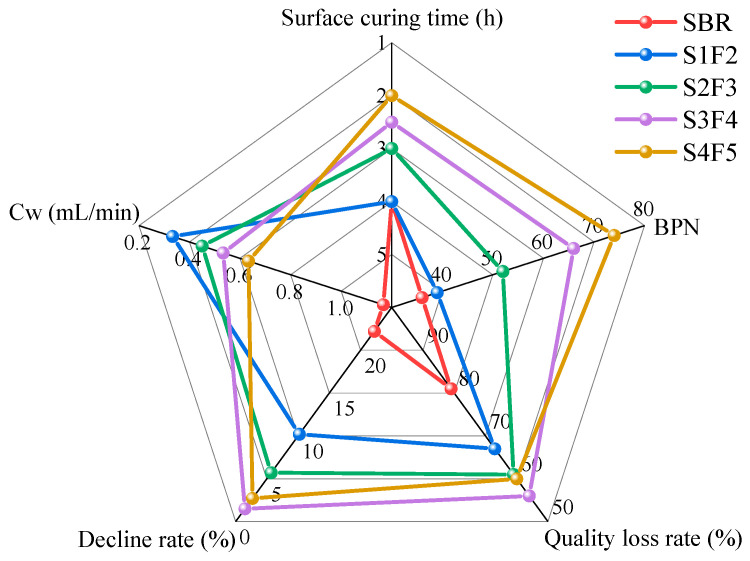
Comparison of different fog seal materials performance.

To more intuitively illustrate the durability performance of different fog seal materials, a comprehensive comparative analysis of the test results is presented in Figure 8. Five indicators were selected to represent key performance aspects: surface curing time (surface curing performance), BPN (skid resistance), seepage coefficient (impermeability property), mass loss rate after 10,000 wear cycles (abrasion resistance), and friction coefficient reduction rate before and after freeze–thaw cycles (freeze–thaw resistance). Figure 8 shows that compared to the SBR-emulsified asphalt fog seal material, the sand-containing fog seal exhibited varying degrees of improvement across all indicators. Higher fine aggregates content and spraying amount can improve the skid resistance, abrasion resistance, freeze–thaw resistance, and reduce the surface curing time of sand-containing fog seal materials. However, excessive fine aggregates and spraying amount adversely affect impermeability performance. S3F4 exhibited excellent performance in terms of abrasion resistance and freeze–thaw resistance, as it showed the lowest mass loss rate (56%) after 10,000 wear cycles and the smallest reduction rate in friction coefficient (1.5%) after freeze–thaw cycles. Moreover, it had a lower permeability coefficient (0.53 mL/min) compared with S4F5. In contrast, S4F5 performed better in skid resistance and surface curing time, showing the highest BPN (74) and the shortest surface curing time (2 h). By comparison, the values of S3F4 for these two indicators were not significantly lower, being 66 and 2.5 h, respectively. Therefore, considering the comprehensive performance indicators, S3F4 (with the spraying amount of 1.0 kg/m^2^ and the fine aggregates content of 30%) is identified as the optimal mix ratio, providing the best overall durability for the sand-containing fog seal materials.

## 4. Conclusions

In this study, a composite modified emulsified asphalt with sand suspension slurry was prepared. The effects of spraying amount and fine aggregates content on abrasion resistance were investigated through dry wheel wear tests and two-way ANOVA. Subsequently, a comprehensive evaluation was conducted from five aspects, surface dryness, skid resistance, wear resistance, impermeability, and freeze–thaw resistance, and finally the optimal mix design of sand-containing fog seal was determined. This study reached the following conclusions:

(1) Under different spraying amounts, the corresponding optimal fine aggregates contents were determined to be 0.8 kg/m^2^ and 20%, 0.9 kg/m^2^ and 25%, 1.0 kg/m^2^ and 30%, and 1.1 kg/m^2^ and 35%. Among these, the combination of 1.0 kg/m^2^ spraying amount and 30% fine aggregates content achieved the best abrasion resistance. Fine aggregates content and spraying amount showed a positive correlation, indicating that these two factors need to be coordinated and optimized to enhance abrasion resistance.
(2) Fine aggregates content has a significant effect on the *WTAT* value, while the spraying amount had no significant effect. However, a significant interaction effect between these two factors was observed (*p* = 0.024). This indicates that simply increasing the spraying amount or fine aggregates content individually cannot further improve the abrasion resistance of the sand-containing fog seal.
(3) After freeze–thaw cycles, the SBR-modified emulsified asphalt showed decreases in BPN and mass loss rate by about 22.2% and 81%, respectively. In contrast, the maximum reductions for the sand-containing fog seal were only 10.2% and 67%, respectively. The sand-containing fog seal materials showed significantly lower mass loss rates, with the 1.0 kg/m^2^ and 30% mix achieving the best abrasion and freeze–thaw resistance.
(4) Compared to the SBR-modified emulsified asphalt, which had a longer surface curing time and lower friction coefficient, the durable sand-fog seal material cured fastest under the 1.1 kg/m^2^ and 35% mix, and the friction coefficient increased by up to 90%. All four optimal sand-containing fog seals demonstrated excellent impermeability, with the 0.8 kg/m^2^ and 20% mix showing the best water sealing performance.
(5) Based on the comprehensive evaluation, the sand-containing fog seal with a spraying amount of 1.0 kg/m^2^ and 30% fine aggregates content demonstrated the best durability. However, the pavement performance of sand-containing fog seal materials applied to airport asphalt pavements may be affected by other additives. Therefore, further research is needed to investigate their influence on the measured parameters.

## Figures and Tables

**Table 1:** Basic properties of emulsified asphalt.

Properties		Test Results	Requirements	Test Methods
Residue by distillation (%)		59.75	≥50	T0651
Surplus on sieve (%)		0.02	≤0.1	T0652
1 day storage stability (%)		0.32	≤1	T0655
5 days storage stability (%)		1.42	≤5	T0655
Asphalt standard viscosity (25 °C) (s)		15.6	8–20	T0621
Evaporated residue	Penetration (25 °C) (0.1 mm)	67.2	50–300	T0604
	Ductility (15 °C) (mm)	59.2	≥50	T0605
	Solubility (%)	99.2	≥97.5	T0607

**Table 2:** Mass ratios of different additives relative to SBR/WER-modified emulsified asphalt.

Components	Fine Aggregates	Thickener	Defoamer	Surfactant	Dispersant	Cement
Mass ratio	15–30%	15%	0.8	1.0	2.0%	3.0%

**Table 3:** Abbreviation of different sand-containing fog seal materials.

Spraying Amount (kg/m^2^)	Fine Aggregates Content (%)				
	15	20	25	30	35
0.8	S1F1	S1F2	S1F3	S1F4	S1F5
0.9	S2F1	S2F2	S2F3	S2F4	S2F5
1.0	S3F1	S3F2	S3F3	S3F4	S3F5
1.1	S4F1	S4F2	S4F3	S4F4	S4F5

**Table 4:** Inter-subject effect test.

	Type III Sum of Squares	Degrees of Freedom	Mean Square	F	Significance (*p*-Value)
Modified model	106,500	19	5600	255.6	0.950
Intercept	5,009,400	1	5,009,400	228,400	0.000
Fine aggregates content	10,200	4	2600	116.3	0.032
Spraying amount	48,100	3	16,000	731.3	0.351
Fine aggregates content and spraying amount	48,200	12	4000	183.0	0.024
Error	1300	60	21.9	-	-
Total	5,117,300	80	-	-	-
Total after correction	107,800	79	-	-	-

**Table 5:** Surface curing time of different fog seal materials.

Materials	SBR-Modified Emulsified Asphalt	S1F2	S2F3	S3F4	S4F5
Surface curing time (h)	4	4	3	2.5	2

**Table 6:** The seepage coefficient of different fog seal materials.

Materials	SBR-Modified Emulsified Asphalt	S1F2	S2F3	S3F4	S4F5
*C_w_* (mL/min)	1.17	0.33	0.45	0.53	0.63

## Data Availability

The original contributions presented in this study are included in the article. Further inquiries can be directed to the corresponding author.
